# VmPacC Is Required for Acidification and Virulence in *Valsa mali*

**DOI:** 10.3389/fmicb.2018.01981

**Published:** 2018-08-23

**Authors:** Yuxing Wu, Zhiyuan Yin, Liangsheng Xu, Hao Feng, Lili Huang

**Affiliations:** State Key Laboratory of Crop Stress Biology for Arid Areas, China–Australia Joint Research Centre for Abiotic and Biotic Stress Management, College of Plant Protection, Northwest A&F University, Yangling, China

**Keywords:** *Malus domestica*, deletion mutant, pH regulation, pectinase, virulence

## Abstract

The role of the transcription factor PacC has been characterised in several pathogenic fungi, and it affects virulence via several mechanisms. In this study, we examined the role of the PacC homolog VmPacC in *Valsa mali*, the causal agent of apple canker disease. We found that the expression of *VmPacC* was up-regulated in neutral and alkaline pH and during infection. At pH 6–10, the radial growth of a *VmPacC* deletion mutant decreased compared to wild-type. In addition, the sensitivity to oxidative stress of the *VmPacC* deletion mutant was impaired, as its growth was more severely inhibited by H_2_O_2_ than that of the wild-type. The lesion size caused by the *VmPacC* deletion mutant was smaller than that of the wild-type on apple leaves and twigs. Interestingly, expression of pectinase genes increased in deletion mutant during infection. To further confirm the negative regulation, we generated dominant activated C-27 allele mutants that constitutively express *VmPacC*. The pectinase activity of activated mutants was reduced at pH 4. We further observed that *V. mali* can acidify the pH during infection, and that the capacity for acidification was impaired after *VmPacC* deletion. Furthermore, VmPacC is involved in the generation of citric acid, which affects virulence. These results indicate that VmPacC is part of the fungal responses to neutral and alkaline pH and oxidative stress. More importantly, VmPacC is required for acidification of its environment and for full virulence in *V. mali*.

## Introduction

To adapt to changing environmental conditions, fungi require an intracellular pH homeostasis system and regulatory mechanism that ensures that directly exposed molecules such as secreted enzymes can function at optimal pH (Penalva and Arst, 2002). An important system to regulate ambient pH in fungi is the Pal signalling pathway. This pathway includes seven components: PalA, PalB, PalC, PalF, PalH, PalI, and PacC. PacC is a transcription factor that regulates pH-dependent gene expression at the end of the pH signalling pathway (Peñalva et al., 2008). In *Aspergillus nidulans*, the PacC polypeptide shows a closed conformation with a processing protease domain inaccessible at acidic conditions. However, the full-length polypeptide is cleaved after a shift to neutral or alkaline conditions, and an intermediate 53 kDa fragment is generated (Díez et al., 2002; Peñalva et al., 2008). The 53-kDa fragment is cleaved again by the proteasome, and results in the final active form of 27-kDa PacC27 (Hervas-Aguilar et al., 2007). In addition, the binding motif of the PacC transcription factor is GCCARG. Transcription of alkaline-expressed genes is activated by PacC at alkaline pH, whereas transcription of acid-expressed genes is repressed at the same condition (Tilburn et al., 1995; Penalva and Espeso, 1996).

In several plant pathogenic fungi, the involvement of PacC in virulence has been demonstrated. Deletion of *PacC* homologs reduces fungal virulence in *Colletotrichum gloeosporioides* (Yakoby et al., 2000), *Penicillium digitatum* (Zhang et al., 2013), *Magnaporthe oryzae* (Landraud et al., 2013), *Fusarium oxysporum* (Caracuel et al., 2003), and *Sclerotinia sclerotiorum* (Rollins, 2003). PacC not only detects environment conditions, but also modifies these conditions via acid or ammonia secretion. Some plant pathogens are therefore grouped into two categories. The first group can alkalinise their host tissue and enhance virulence by producing ammonia. Examples include *Colletotrichum* spp. and *Alternaria alternata* (Eshel et al., 2002; Alkan et al., 2009). The other group of pathogens, including *Penicillium* spp., *Botrytis cinerea*, and *S. sclerotiorum*, can acidify the host tissue by producing acids (Wubben et al., 2000; Rollins and Dickman, 2001; Barad et al., 2012; Zhang et al., 2013). This modulation of host environment provides a suitable condition for the function of virulence factors like secreted enzymes. It may also serve as a signal to activate the production of these virulence factors. In addition to modify environment pH, PacC is also known to regulate some cell wall degrading enzymes such as polygalacturonase (Rollins, 2003), pectate lyase (Kramer-Haimovich et al., 2006), and endoglucanase to affect fungal virulence (Eshel et al., 2002).

Apple Valsa canker is a bark disease of apple trees (*Malus* sp.) that seriously imperils apple production in China (Wang et al., 2014), Korea (Lee et al., 2006), and Japan (Abe et al., 2007). This disease is caused by the parasite fungus *Valsa mali. V. mali* infects the bark of apple trees through various wounds such as pruning ends, sunscalds, frostbites, and some mechanical injuries (Ke et al., 2013). Following colonisation of the wounded tissue, the hyphae spread to all bark tissues, resulting in severe tissue maceration and necrosis. As a plant pathogenic fungus without specialised penetration structures, *V. mali* secretes hydrolytic enzymes, particularly pectinases, involved in disease development and pathogenesis (Ke et al., 2013, 2014; Feng et al., 2017b). Pectinases are considered virulence factors because the virulence of several *V. mali* pectinase gene deletion mutants is attenuated (Yin et al., 2015). However, these findings do not clarify the role of PacC in the regulation of virulence in this important woody plant fungal pathogens. Understanding the role of PacC during *V. mali* infection might allow the development of novel sustainable apple Valsa canker management strategies. In the present study, we have investigated the role of PacC in pH acidification, oxidative stress, and pectinase gene expression during *V. mali* infection.

## Materials and Methods

### Strains and Culture Conditions

The wild-type strain of *V. mali* named 03-8 (Laboratory of Integrated Management of Plant Diseases in College of Plant Protection, Northwest A&F University) was used (Yin et al., 2015). All strains including the wild-type and the transformants generated in this work were propagated on potato dextrose agar (PDA, 20% potato extract, 2% dextrose, 1.5% agar) when necessary. Potato dextrose broth (PDB, 20% potato extract, 2% dextrose) was used for measuring the dynamic pH change or for liquid culture.

Growth of wild-type and mutant strains on PDA media at different pH were assayed by measuring colony diameters 2 days post-inoculation (dpi) at 25°C. PDA medium was adjusted with HCl or NaOH to pH 3–10. The PDA media mended NaCl (0.1 M), H_2_O_2_ (3 mM), congo red (CR, 300 mg/L), or sodium dodecyl sulfonate (SDS 0.01%) were used for stress response assays. For selection of transformants during gene deletion, TB_3_ medium (0.3% yeast extract, 0.3% casamino acids, 20% sucrose, 1.5% agar) supplemented with 100 μg/mL hygromycin B (Calbiochem, La Jolla, CA, United States) or 100 μg/mL geneticin (Sigma, St. Louis, MO, United States) was used. To determine protein concentration and pectinase activity, synthetic medium (SM) (Srivastava et al., 2012) with 1% pectin as the sole carbon source was used. Buffered SM medium was obtained using 0.05 M Na_2_HPO_4_ and 0.05 M C_4_H_2_O_7_ to maintain pH 4 (38.5% volume ratio of Na_2_HPO_4_) or pH 7 (82.4% volume ratio of Na_2_HPO_4_).

### Twig and Leave Infection Assays

Wild-type and mutant strains were cultured on PDA for 2 days. Five-mm agar plugs were taken from the edge of a colony and were inoculated in the armature wound on the fourth or fifth leave from the top of a branch at 25°C for 3 days. Lesion diameters were recorded. For twig assays, the agar plugs were inoculated in the scald wound on 1-year old twigs of *Malus domestica* borkh. ‘Fuji’ at 25°C for 9 days. The lesion lengths were recorded (Wu et al., 2017b).

### Nucleic Acid Isolation and Manipulation

*VmPacC* genes were originally identified through homology searches in the *V. mali* genomic sequence (Accession No. JUIY00000000.1) using the PacC amino acid sequence of *A. nidulans* as query (Q002.2.1). The neighbour-joining method was used to construct phylogenetic trees of Pal pathway proteins (Tamura et al., 2007). **Supplementary Table S1** shows the pectinase genes annotated in the *V. mali* genome.

Gene disruption constructs were generated by replacing the complete open reading frame (ORF) of *VmPacC* gene (**Supplementary Figure S2A**). The primer pairs VmPacC-1F/2R and VmPacC-3F/4R were used to amplify the upstream and downstream flanking sequences, respectively, of the target gene (primer sequences are shown in **Supplementary Table S2**). The hygromycin resistance gene (*hph*) fragment was amplified with the primer pair HYG/F and HYG/R. Deletion cassettes were constructed using double-joint PCR as described (Yu et al., 2004). The deletion cassettes were transformed into protoplasts of the 03-8 strain using the polyethylene glycol (PEG) method as described (Gao et al., 2011). Following screening in hygromycin-containing medium, the transformants were tested using PCR with primer pairs VmPacC-5F/6R, VmPacC-7F/H855R, or H856F/VmPacC-8R to confirm gene replacement events (**Supplementary Table S2**). Putative gene deletion mutants were further confirmed using Southern blotting with target (probe p) and hygromycin (probe h) gene probes and the DIG-High Prime DNA Labelling and Detection Starter Kit II (Roche, Penzberg, Germany).

Complementation mutants and activated mutants were generated using the gap repair approach by co-transformation of *VmPacC* gene or activated fragments (**Supplementary Figure S3A**) amplified with primer pairs VmPacC-C (27)-F/R and *Cla*I-digested plasmid pFL2 into yeast strain XK1-25 (Zhou et al., 2012; Wu et al., 2017a). Trp^+^ yeast transformants were screened for the desired fusion constructs, which were confirmed by sequencing and transformed into the *VmPacC* deletion mutant. Geneticin-resistant transformants of complementation with the desired constructs were identified using PCR with primer pairs VmPacC-C-F/R and Southern blotting. The activated geneticin-resistant transformants were detected using PCR with primer pairs VmPacC27-F/R (**Supplementary Figure S3B**). The activated mutants were further identified by measuring the growth at pH 9 in unbuffered PDA for 3 days.

Transcript levels of *VmPacC* and pectinase-related genes were determined using real-time PCR. For expression of *VmPacC* in different pH, strains were cultured on PDA for 2 days. Five-mm agar plugs were taken from the edge of a colony and were cultured in a non-buffered PDB medium (pH 5.5) for 48 h. Mycelia were then transferred to fresh media buffered to pH 4, pH 7, or pH 9 for 6 h. The colonised apple tree bark was sampled as described (Ke et al., 2014). RNA was isolated with the TRIzol reagent (Invitrogen, Carlsbad, CA, United States) as described (Yin et al., 2013). For qRT-PCR assays, we used the Fermentas (Hanover, MD, United States) 1^st^ strand cDNA synthesis kit following instructions of the manufacturer. The glyceraldehyde-6-phosphate dehydrogenase (*G6PDH*) gene of *V. mali* was used as internal control (Yin et al., 2013). Relative transcript levels of each gene were calculated by the 2^-ΔΔC_T_^ method (Livak and Schmittgen, 2001). Data from three replicates were used to calculate means and standard deviations. Statistical analysis was done using the Student’s *t*-test implemented in the SAS software package (SAS Institute), *P* < 0.05. Primers used for gene expression are listed in **Supplementary Table S2**.

### Pectinase Activity and Protein Concentration Assays

Five-mm agar plugs were taken from the edge of a colony and were cultured in PDB medium for 48 h. Mycelia were transferred to SM buffered to pH 4 or pH 7 and supplemented with 1% pectin as the sole carbon source for 12 h. Pectinase activity was quantified using the 3,5-dinitrosalicylic acid (DNS) method as described (Wu et al., 2017b).

Total extracellular protein content in culture supernatant was measured using the Bradford Protein Assay Kit (TIANGEN, China) with absorbance at 595 nm, and using bovine serum albumin as the standard.

### pH and Citric Acid Measurements

The pH value of liquid PDB was measured with a pH electrode (Mettler Toledo, Shanghai, China) at different times after incubation. The pH of inoculation sites on the apple tree bark was measured using a flathead pH electrode as described (Schmidt et al., 2001). Three replicates were tested for each treatment.

High-performance liquid chromatography (HPLC) was used to determine citric acid content (Chinnici et al., 2005). Five-mm agar plugs were taken from the edge of a colony and were cultured in PDB medium for 48 h. Mycelia were transferred to SM supplemented with 0.5% saccharose as the sole carbon source for 48 h. The supernatant was filtered through a 0.22 μm cellulose acetate membrane before injection and measured using ion-exclusion chromatography. A Waters 600E HPLC apparatus with an attached refractive index detector (2414 RI) was used. Samples were separated in a Sepax Carbomix H-NP column under the following conditions: 2.5 mM H_2_SO_4_ mobile phase, 55°C column temperature, 0.6 mL/min flow-rate, and 30 min or less of analysis time. Peak data were collected with Empower LC solution. Citric acid standard (Solarbio, China) at 0.01–1 mg/mL was used for the standard curve.

## Results

### Gene Expression Analysis of *VmPacC* in *V. mali*

The *V. mali* genome contains single copy genes for pH-signalling pathway proteins designated VmPalA, VmPalB, VmPalC, VmPalF, VmPalI, VmPalH, and VmPacC. Analysis of the amino acid sequences revealed significant similarities to four fungi pH signalling pathway protein sequences (**Supplementary Figure S1A**). *VmPacC* codes for 560 amino acid residues interrupted by two introns, being smaller than the *PacC/Rim101* orthologs of *A. nidulans* (678 aa) and yeast (635 aa). In addition, *VmPacC* contains classical zinc-finger domain and Zinc-finger double domains at its N-terminal region (residues 116–138 and 102–127) (**Supplementary Figure S1B**).

**FIGURE 1:**
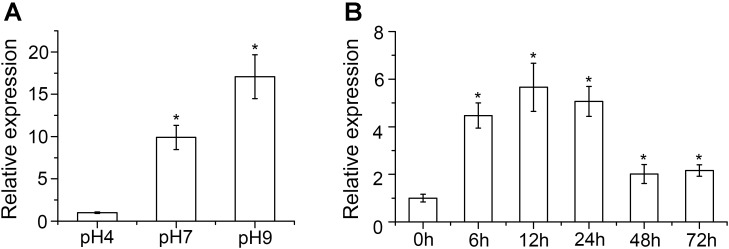
Expression profiles of *VmPacC* in *Valsa mali*. **(A)** Quantitative real-time PCR analysis of *VmPacC* expression in fungus grown at different pH conditions. RNA samples were isolated from mycelia of the wild-type strain on potato dextrose broth (PDB) medium at pH 4, 7, and 9 after pre-growth on unbuffered PDB for 48 h. **(B)** Quantitative real-time PCR analysis of *VmPacC* expression during infection. RNA samples were isolated from apple tree barks around the inoculation site at 0, 6, 12, 24, 48, and 72 h post-inoculation. Transcript abundances (mean and standard deviation) were calculated using data from three independent biological replicates. The asterisk represents a statistically significant difference (*P* < 0.05).

Expression of *VmPacC* in different pH and infection conditions was determined. After transferring to different pH for 6 h, qRT-PCR analysis showed that *VmPacC* gene expression was upregulated 9.9- and 17-fold in pH 7 and pH 9, respectively, compared to that of pH 4 (**Figure 1A**). These results show the important regulatory role of this gene in alkaline conditions. Upregulated *VmPacC* expression was also observed during infection. After inoculating *V. mali* in 1 year-old twigs, the *VmPacC* gene expression increased and reached a peak 12 h post-inoculation, and then decreased and remained stable at twofold (**Figure 1B**). These results suggest that VmPacC might be involved in *V. mali* virulence.

### VmPacC Is Required for Mycelial Growth at Alkaline pH

To investigate the roles of *VmPacC* genes in *V. mali*, we generated a deletion mutant in which the entire ORF of *VmPacC* was deleted. We constructed a gene replacement cassette by placing upstream and downstream flanking sequences next to *hph* resistance gene sequences (**Supplementary Figure S2A**). The cassette was introduced in protoplasts of the wild-type strain. Three *VmPacC* deletion mutants (*ΔVmPacC*) were obtained from 215 hygromycin-resistant transformants and identified using PCR (**Supplementary Figure S2B**). When hybridised with the *VmPacC* ORF probe (probe p), a 5.6-kb *Cla*I fragment was detected in the wild-type strain but not in the *ΔVmPacC* mutant. In addition, *ΔVmPacC* showed a single locus homologous recombination event after hybridisation with the hygromycin probe (probe h) (**Supplementary Figure S2C**). Complementation mutant strains (*ΔVmPacC-C*) were also generated by re-introducing a wild-type allele of *VmPacC* in deletion mutants at an ectopic locus.

**FIGURE 2:**
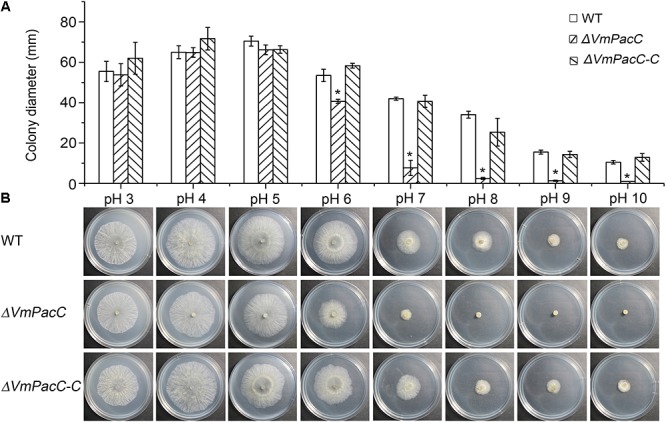
*VmPacC* is required for adaptation to ambient pH in *V. mali*. **(A)** Quantification of growth rate incubated for 2 days at 25°C. Results are the average of three independent experiments and standard deviations are shown. The asterisk represents a statistically significant difference compared to wild-type (*P* < 0.05). **(B)** Growth of wild-type, *VmPacC* deletion, and complemented mutant strains in unbuffered potato dextrose agar (PDA) adjusted to pH 3 to pH 10 with HCl or NaOH.

Wild-type, *ΔVmPacC*, and *ΔVmPacC-C* strains were grown on unbuffered PDA at pH 3 to pH 10. The wild-type strain of *V. mali* showed a more wide-range adaptation to pH 3 to pH 10. The colony diameters of the *ΔVmPacC* strain on PDA were similar to those of the wild-type at pH 3 to pH 5, but were reduced at pH 6 to pH 10. The deletion mutant hardly grew at pH 8 or higher (**Figure 2**). Normal growth was restored in the *ΔVmPacC-C* mutant, indicating that VmPacC regulates growth in alkaline conditions.

### VmPacC Is Involved in the Oxidative Stress Response

**FIGURE 3:**
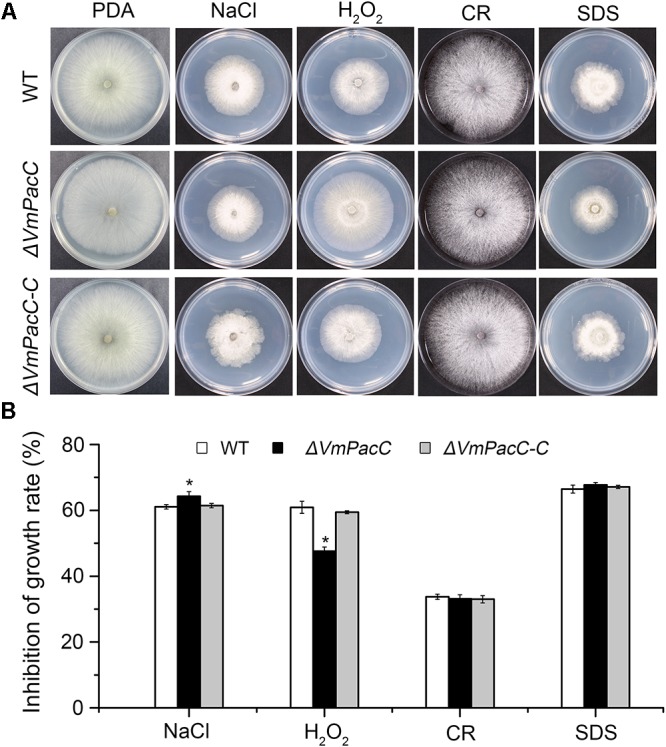
Effects of inactivation of *VmPacC* gene on the response of *V. mali* to stress. **(A)** Cultures of the wild-type, *VmPacC* gene null mutant, and complemented strains grown on PDA supplemented with 1 M sorbitol, 0.1 M NaCl, 3 mM H_2_O_2_, 300 mg/L Congo red (CR), or 0.01% SDS. Images were taken after 2.5 days on PDA and 3.5 days in inhibitor medium. **(B)** Inhibition of growth (colony diameter) on PDA supplemented with inhibitor compared with PDA without stress. The asterisk represents a statistically significant difference compared to wild-type (*P* < 0.05). Bars indicate standard deviations of the mean of three replicates.

To test whether VmPacC is involved in the response to abiotic stress, we measured the colony diameters of wild-type, *ΔVmPacC*, and *ΔVmPacC-C* strains on PDA supplemented with NaCl, H_2_O_2_, CR, or SDS. Results showed that the growth inhibition of NaCl on the *ΔVmPacC* mutant was higher than that on the wild-type or complemented mutant strains. However, the sensitivity of the VmPacC deletion mutant was reduced on PDA supplemented with H_2_O_2_. In addition, no significant differences were observed between wild-type, *ΔVmPacC*, and *ΔVmPacC-C* on PDA supplemented with CR or SDS (**Figure 3**). These results suggest that VmPacC is involved in the response to oxidative stress.

### VmPacC Is Required for Virulence

**FIGURE 4:**
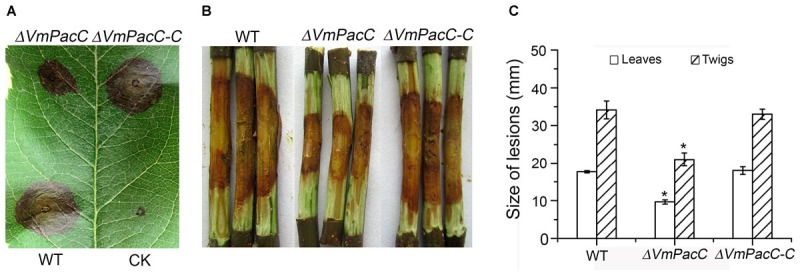
Phenotype of leaves and twigs inoculated with *VmPacC* deleted mutants of *V. mali*. **(A)** Apple leaves were inoculated with mycelium agar plugs from the wild-type, deletion mutant, and complemented strains. Lesion sizes are shown at 3 days post-inoculation (dpi). **(B)** Apple twigs were scald inoculated with mycelium agar plugs from the wild-type, deletion mutant, and complemented mutant strains. Lesion sizes are shown at 9 dpi. **(C)** Quantitation of the lesion size on apple leaves is lesion diameter at 3 dpi. Quantitation of the lesion size on twigs is lesion length at 9 dpi. The asterisks represent a statistically significant difference compared to wild-type (*P* < 0.05). Bars indicate standard deviations of the mean of three replicates.

To determine whether VmPacC plays an important role in disease development, virulence assays were performed on leaves and twigs using the wild-type, *ΔVmPacC*, and *ΔVmPacC-C* strains. The diameter of the lesions in leaves inoculated with the wild-type was 34 mm at 3 dpi, whereas that of *ΔVmPacC*-inoculated leaves was 17 mm. When twigs were used as host, the length of lesions was also smaller in samples infected with the *ΔVmPacC* strain. In addition, infection with the complementation strains restored the wild-type phenotype (**Figure 4**). These results clearly indicate that VmPacC plays a significant role in controlling virulence in *V. mali*.

### VmPacC Suppresses the Production of Pectinase

**FIGURE 5:**
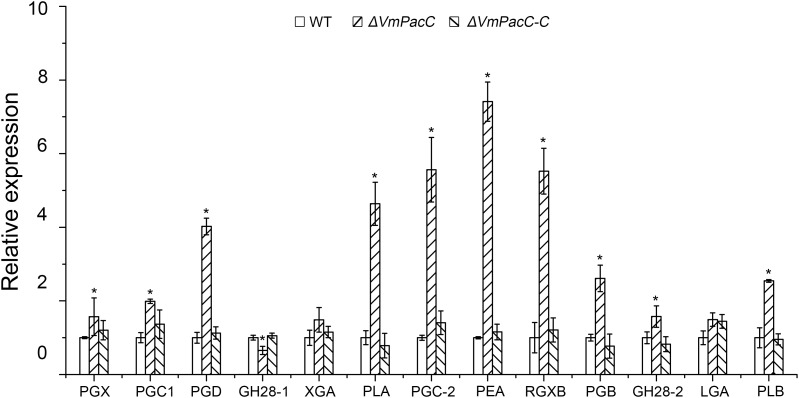
Expression levels of pectinase genes in wild-type, deletion mutant, and complemented mutant strains determined by qRT-PCR. RNA samples were isolated from lesion borders of apple tree bark at 3 days post-inoculation. Wild-type expression levels were arbitrarily set to 1. The mean was calculated using data from three independent biological replicates. The asterisk represents a statistically significant difference compared to wild-type.

To further evaluate the role of VmPacC on the virulence factor pectinase, we determined the transcript levels of a dozen pectinase genes in the deletion mutant and compared to those of the wild-type strain in colonised apple tree bark. Unexpectedly, transcript levels of most pectinase genes increased to different degrees (**Figure 5**). These results suggest that VmPacC negatively regulates pectinase expression.

**FIGURE 6:**
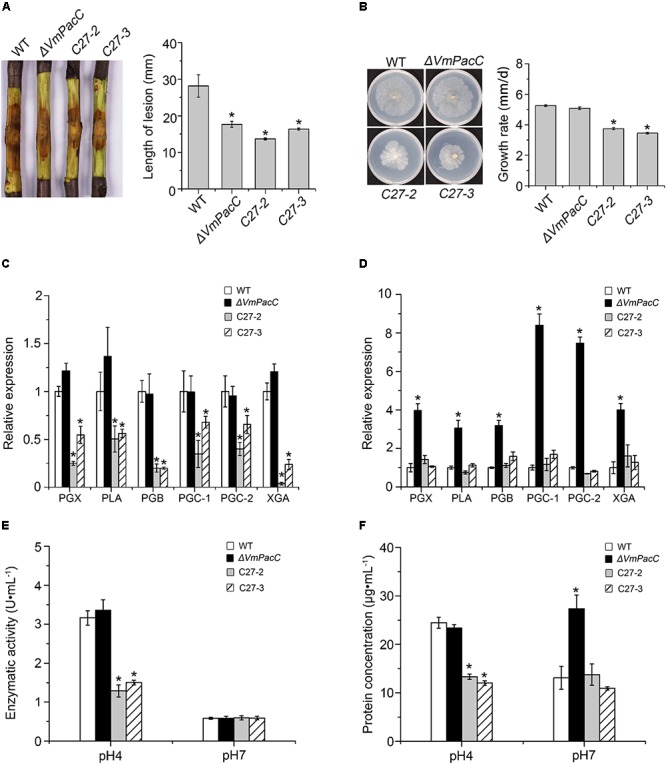
Negative regulation of pectinase production by *VmPacC* dominant activated mutants C27-2 and C27-3. **(A)** Phenotypes of twigs inoculated with wild-type, *VmPacC* deletion mutant, and activated mutants C27-2 and C27-3; length of lesions was measured at 9 days post-inoculation. **(B)** Mycelial growth on SM supplemented with pectin for 6 days. Expression levels of pectinase genes in wild-type, deletion mutant, and activated mutant strains cultured in SM medium supplemented with pectin at pH 4 **(C)** and pH 7 **(D)** determined using qRT-PCR. **(E)** Pectinase activity of different strains cultured in MS supplemented pectin for 12 h. **(F)** Assays for protein concentration of wild-type, deletion mutant, and activated mutant strains in 1% pectin inducing medium after culture for 12 h. Bars marked by asterisks in each group differ significantly from wild-type (LSD, *P* < 0.05). Error bars represent standard deviations from three replicates.

To further confirm the repressive effect of VmPacC on pectinase expression, we generated dominant activated allele mutants, strains C-27, which constitutively express VmPacC in an acid environment. C-27 strains showed normal growth compared to wild-type on alkaline PDA indicating the VmPacC was expressive (**Supplementary Figure S3C**). However, C-27 strains showed significantly reduced virulence (**Figure 6A**). When inoculated on solid medium with pectin as the sole carbon source, the growth rate of C-27 was reduced compared with the wild-type and *ΔVmPacC* strains (**Figure 6B**). To determine the requirement of VmPacC for pH-dependent expression of pectinase genes, pectinase transcript levels and activity in the wild-type, *ΔVmPacC*, and C-27 strains were assayed (**Figures 6C,D**). The results show that pectinase activity in the supernatant of C-27 strains was reduced compared to that of wild-type and *ΔVmPacC* strains at pH 4 (**Figure 6E**). At pH 7, the pectinase genes of *ΔVmPacC* were up regulated 3- to 7-fold, and protein content was also increased (**Figure 6F**). However, pectinase activity was similar in all strains possible because the optimum pH value of pectinases is 3.5 (**Figure 6E**) (Feng et al., 2017a). These results indicate that pectinases are suppressed by VmPacC.

### VmPacC Is Required for Acidification of the *V. mali* Environment

**FIGURE 7:**
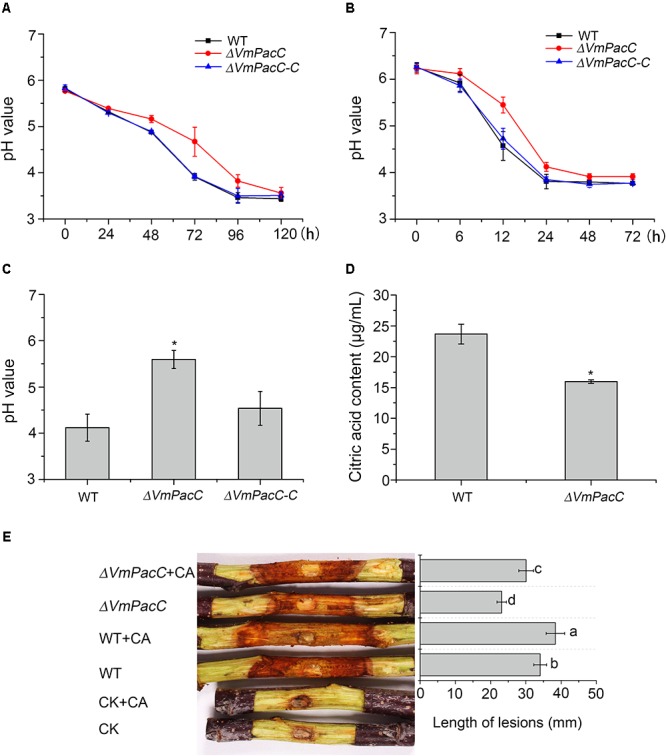
*Valsa mali* modulates its environmental pH. **(A)** Decrease in pH value during growth of different strains for 120 h. **(B)** pH values at the inoculated sites in the apple tree bark. **(C)** pH values at the lesion borders of the apple tree bark. **(D)** Citric acid production in wild-type and *VmPacC* deletion mutant strains grown in PDB determined by high-performance liquid chromatography (HPLC). The experiments were repeated three times. The asterisk represents a statistically significant difference compared to wild-type. **(E)** Virulence assays performed on twigs with wild-type and deletion mutant strains using citric acid (CA). The length of lesions was measured at 9 days post-inoculation. The experiments were repeated three times. Different letters represent a significant difference (*P* < 0.05).

To investigate the involvement of VmPacC in the modification of the environment, the dynamic change in pH of the wild-type, *ΔVmPacC*, and *ΔVmPacC-C* strains was measured in an unbuffered liquid medium. The pH value of the wild-type strain decreased from the initial pH 5.8 to pH 3.4 in 120 h. Even though the same decrease in pH units was observed after 96 h in the *VmPacC* deletion mutant, the rate of descent was slower than in the wild-type (**Figure 7A**).

When the wild-type strain of *V. mali* was inoculated in detached apple twigs, the initial pH at the inoculation sites was 6.2. The pH decreased to 3.8 when canker symptoms appeared 24 h after inoculation. Thereafter, the pH remained relatively stable. However, the same pH value (pH 3.9) was determined 48 h after inoculation in the *VmPacC* deletion mutant. The pH value of the deletion mutant decreased more slowly than in the wild-type strain. Furthermore, the pH value at the border of the lesion in the deletion mutant was higher than in the wild-type strain. The acidification capacity was impaired when *VmPacC* was deleted, and this effect could be reversed by complementation of the deletions (**Figures 7B,C**).

High-performance liquid chromatography analysis of organic acids secreted by *V. mali* indicated that the accumulation of citric acid was reduced in the *VmPacC* deletion mutant compared to wild-type (**Figure 7D**). To further test whether acidification of the environment contributes to virulence, we have inoculated the deletion mutant and externally treated with citric acid at pH 3.5. As expected, the virulence of the *VmPacC* deletion mutant and the wild-type strain increased when externally treated with citric acid (**Figure 7E**).

Our results clearly show that *V. mali* requires acidification of its environment for growth. In addition, *Vm*PacC is involved in virulence partly via regulating the generation of citric acid.

## Discussion

The Pal ambient pH response signalling pathways are essential for fungi to adapt to environmental and host conditions. This signalling cascade system ultimately activates the PacC transcription factor, which controls the environmental pH sensing and response (Peñalva et al., 2008). In this study, we found that the optimum pH of the apple bark pathogenic fungus, *V. mali*, is acid. Neutral or alkaline conditions did not favour mycelial growth, as indicated by the impaired vegetative growth of deletion mutant *VmPacC* at neutral and alkaline conditions (**Figures 2A,B**). These results indicate that VmPacC is required for *V. mali* adaptation to alkaline pH. This is consistent with what has been described in other fungi such as *P. digitatum* (Zhang et al., 2013), *S. sclerotiorum* (Rollins, 2003), *F. graminearum* (Merhej et al., 2011), and *A. nidulans* (Tilburn et al., 1995). In contrast, in *Ustilago maydis*, the null rim101 mutants show normal growth rate *in vitro*(Arechiga-Carvajal and Ruiz-Herrera, 2005). Moreover, in *Metarhizium robertsii* and *M. oryzae*, PacC affects growth under both acidic and alkaline conditions (Landraud et al., 2013; Huang et al., 2015). These results suggest that, in addition to adaptation to alkaline pH, alternative PacC signalling pathways may have evolved in some fungi.

We also found that VmPacC affected the sensitivity to salt and oxidative stress in *V. mali* (**Figure 3**). Similarly, deletion of PacC/Rim101 reduces ion tolerance because of repression of ion pump genes (Caracuel et al., 2003). In the insect pathogenic fungi *M. robertsii*, the reduced ion tolerance in *ΔMrpacC* results from smaller vacuoles (Huang et al., 2015). In pathogen–host interactions, the reactive oxygen species (ROS) play an important role. Plants use the oxidative burst as an early defence reaction to pathogen attack. Here, we show that *Vm*PacC is involved in the sensitivity to oxidative stress. However, this issue is indeterminacy in necrotrophs. For example, in *B. cinerea*, deletion of the AP-1 transcription factor gene does not lead to impaired virulence (Temme and Tudzynski, 2009). Thus, for *V. mali*, the relation between abiotic stress responses and virulence needs further research.

The present study showed that deletion of the VmPacC gene significantly impaired the virulence of *V. mali* (**Figure 4**). Similar to what has been reported for PacC mutant strains of *P. digitatum* (Zhang et al., 2013), *S. sclerotiorum* (Rollins, 2003), *C. gloeosporioides* (Miyara et al., 2008), and *M. oryzae* (Landraud et al., 2013), PacC regulates the virulence in a positive manner in *V. mali* (**Figure 3**). However, PacC is a negative regulator of virulence in *F. oxysporum* and *F. graminearum* (Caracuel et al., 2003; Merhej et al., 2011). In addition, the pathogenicity of null rim101 mutants of *U. maydis* is not affected (Arechiga-Carvajal and Ruiz-Herrera, 2005). In phytopathogenic fungi, cell wall-degrading enzymes are important virulence factors during plant infection (Kubicek et al., 2014). The PacC is also a key regulator of secretion of these hydrolytic enzymes in several filamentous fungi (Miyara et al., 2010). In *C. gloeosporioides*, the reduced expression of a pectate lyase gene decreases virulence in a *CgpacC*-disrupted mutant (Miyara et al., 2008). In *P. digitatum*, the expression of cell wall degradation enzyme genes such as *Pdpg2* and *Pdpnl1* is regulated by PdPacC. In addition, this PdPacC regulation is associated with pathogenesis (Zhang et al., 2013). In *S. sclerotiorum*, the induced virulence of a PacC deletion mutant results from impaired expression of endopolygalacturonase (*pg1*) in higher ambient pH (Rollins, 2003). Pectinase is an important virulence factor in *V. mali* (Ke et al., 2013; Yin et al., 2015). Therefore, we determined the transcript levels of six pathogenesis-related pectinase genes and six up-regulated genes during infection (Yin et al., 2015). However, unexpectedly, the expression of most pectinase genes tested was up-regulated to different degrees in *VmPacC* deletion mutants (**Figure 5**). These results show that VmPacC is involved in repressing pectinase genes. This regulation model of virulence and pectinase expression is distinct from the above-mentioned mechanism. Therefore, VmPacC should not impact virulence via regulating the expression of pectinase genes.

To further investigate the repressive effect of VmPacC on pectinase gene expression, we generated dominant activated allele mutants. The mutants constitutively express PacC independent of pH. In agreement with our hypothesis, we found that dominant activated allele mutants showed significantly reduced growth on solid media supplemented with pectin as the sole carbon source. In addition, both the expression and activity of pectinase genes decreased and led to a reduction in the size of lesions on twigs. These results are consistent with the expression of two polygalacturonase genes, *pg1* and *pg5*, being negatively regulated by PacC in *F. oxysporum*, although PacC functions as a negative regulator of virulence in this organism (Caracuel et al., 2003). In several fungi, PacC is responsible for up-regulation of alkaline-expressed genes and suppression of acid-expressed genes in alkaline pH (Alkan et al., 2013). Proteins need to be selectively expressed at the optimal external host pH environment to function correctly. We have demonstrated that the optimum pH value of pectinase activity is 3.5 (Feng et al., 2017a). The pectinase gene should be inhibited by PacC in neutral and alkaline conditions. Although transcript and protein levels in *ΔVmPacC* significantly increase at pH 7, the pectinase activity was the same as in the wild-type. These results indicate that the secreted pectinases do not contribute to pectinase activity in neutral and alkaline conditions.

In plant pathogens, the capacity to modify the ambient pH seems to be associated with infection of the host plants. Several phytopathogenic fungi acidify or alkalinise their surrounding pH during infection. Some plant pathogenic fungi such as *S. sclerotiorum* and *P. digitatum* are known to acidify their environment during infection via the secretion of oxalic acid or other organic acids (Rollins, 2003; Zhang et al., 2013). Other fungi, such as *M. oryzae* and *Colletotrichum* species, tend to secrete ammonia to alkalinise the invaded plant tissues (Landraud et al., 2013; Ment et al., 2015). In the present study, *V. mali* showed similar behaviour to *S. sclerotiorum* and *P. digitatum*. *V. mali* can acidify the surrounding or host pH, and the acidification capacity was impaired after deletion of *VmPacC*. We further demonstrated that VmPacC is required for the accumulation of citric acid in *V. mali*, and that the virulence of the wild-type strain and deletion mutant can be increased by externally adding citric acid to twigs. These results show that VmPacC is involved in virulence partly owing to the regulation of citric acid generation. However, whether citric acid contributes to virulence via another pathway remains to be elucidated.

## Conclusion

We determined that the optimum pH of *V. mali* is acid, and that this organism can acidify its surrounding pH. In addition, the capacity to acidify the environmental pH is regulated by VmPacC, which is also involved in the response to oxidative stress and modulates expression of pectinase genes. More importantly, VmPacC is involved in virulence partly owing to the regulation of citric acid generation. In the future, comparison of the transcriptomes of the wild-type strain and the *VmPacC* deletion mutant of *V. mali* at different pH or during infection of apple bark will contribute to a better understanding of pathways involved in PacC-related pathogenesis.

## Author Contributions

YW and LH conceived and designed the experiments and wrote the paper. YW and LX performed the experiments. YW, ZY, and HF analyzed the experiment data. ZY and LH contributed reagents, materials, and analysis tools. All authors have read and approved the final manuscript.

## Conflict of Interest Statement

The authors declare that the research was conducted in the absence of any commercial or financial relationships that could be construed as a potential conflict of interest.

## Supplementary Material

FIGURE S1 Structure and sequence analysis of pH-signalling pathway proteins in *Valsa mali*. **(A)** Phylogenetic analysis of pH-signalling pathway proteins of *V. mali* and their homologs from *Magnaporthe oryzae*, *Aspergillus nidulans*, and *Neurospora crassa*. The amino acid sequences of Pal pathway proteins were analysed using MEGA version 6 and neighbour-joining analysis with 1,000 bootstrap replicates. Numbers on the branches represent the percentage of replicates supporting each branch. Subclades containing pH-signalling pathway proteins of *V. mali* and orthologs from other species are shaded. The bar represents 20% sequence divergence. **(B)** The open reading frame (ORF) of VmPacC consists of 1,825 bp, interrupted by two introns, and encodes a predicted protein of 560 amino acids with classical zinc-finger domain and zinc-finger double domain at its N-terminal region.

FIGURE S2 Generation of *VmPacC* gene deletion mutants. **(A)** Generation of gene replacement constructs. The small arrows mark the position and direction of primers used for PCR. **(B)** For PCR detection of deletion transformants, four primer pairs (VmPacC-5F/6R, H850F/H852R, VmPacC-7F/H855F, and H856F/VmPacC-8R) were used to detect *VmPacC* gene deletion mutants. **(C)** Southern blots of *Cla*I-digested genomic DNA of wild-type, deletion mutant (*ΔVmPacC*), and complemented mutant (*ΔVmPacC-C*) strains hybridised with probe p (*VmPacC* ORF or probe h (*hph* ORF)).

FIGURE S3 Construction of dominant activated mutant of *VmPacC27*. **(A)** Prediction of activated sequence with *A. nidulans*. **(B)** PCR detection of positive transformants of activated mutants with primer pairs VmPacC27-F/R. **(C)** Wild-type (WT) deletion and activated mutants grow at pH 9 in unbuffered potato dextrose agar for 3 days. The normal growth was restored in the activated mutants, indicating the VmPacC was expressive.

TABLE S1 Pectinase genes for expression.

TABLE S2 Primers used for deletion, complementation, and gene expression.

## References

[B1] Abe K. Kotoda N. Kato H. Soejima J. (2007). Resistance sources to Valsa canker (*Valsa ceratosperma*) in a germplasm collection of diverse Malus species. *Plant Breed.* 126 449–453. 10.1111/j.1439-0523.2007.01379.x

[B2] Alkan N. Davydov O. Sagi M. Fluhr R. Prusky D. (2009). Ammonium secretion by *Colletotrichum coccodes* activates host NADPH oxidase activity enhancing host cell death and fungal virulence in tomato fruits. *Mol. Plant Microbe Interact.* 22 1484–1491. 10.1094/MPMI-22-12-1484 19888814

[B3] Alkan N. Meng X. Friedlander G. Reuveni E. Sukno S. Sherman A. (2013). Global aspects of pacC regulation of pathogenicity genes in *Colletotrichum gloeosporioides* as revealed by transcriptome analysis. *Mol. Plant Microbe Interact.* 26 1345–1358. 10.1094/MPMI-03-13-0080-R 23902260

[B4] Arechiga-Carvajal E. T. Ruiz-Herrera J. (2005). The RIM101/pacC homologue from the basidiomycete *Ustilago maydis* is functional in multiple pH-sensitive phenomena. *Eukaryot. Cell* 4 999–1008. 10.1128/EC.4.6.999-1008.2005 15947192 PMC1151993

[B5] Barad S. Horowitz S. B. Moscovitz O. Lichter A. Sherman A. Prusky D. (2012). A *Penicillium expansum* glucose oxidase–encoding gene, GOX 2, is essential for gluconic acid production and acidification during colonization of deciduous fruit. *Mol. Plant Microbe Interact.* 25 779–788. 10.1094/MPMI-01-12-0002 22352719

[B6] Caracuel Z. Casanova C. Roncero M. I. G. Di Pietro A. Ramos J. (2003). pH Response transcription factor PacC controls salt stress tolerance and expression of the P-Type Na+-ATPase Ena1 in *Fusarium oxysporum*. *Eukaryot. Cell* 2 1246–1252. 10.1128/ec.2.6.1246-1252.2003 14665459 PMC326653

[B7] Chinnici F. Spinabelli U. Riponi C. Amati A. (2005). Optimization of the determination of organic acids and sugars in fruit juices by ion-exclusion liquid chromatography. *J. Food Compos. Anal.* 18 121–130. 10.1016/j.jfca.2004.01.005

[B8] Díez E. Álvaro J. Espeso E. A. Rainbow L. Suárez T. Tilburn J. (2002). Activation of the Aspergillus PacC zinc finger transcription factor requires two proteolytic steps. *Embo. J.* 21 1350–1359. 10.1093/emboj/21.6.1350 11889040 PMC125927

[B9] Eshel D. Miyara I. Ailing T. Dinoor A. Prusky D. (2002). pH regulates endoglucanase expression and virulence of *Alternaria alternata* in persimmon fruit. *Mol. Plant Microbe Interact.* 15 774–779. 10.1094/MPMI.2002.15.8.774 12182334

[B10] Feng H. He Y. Zhen W. Gao X. Wang H. Huang L. (2017a). Isolation, purification and characterization of extracellular pectinase produced by *Valsa mali*. *Microbiol. China* 44 639–647. 10.13344/j.microbiol.china.160236

[B11] Feng H. Xu M. Liu Y. Dong R. Gao X. Huang L. (2017b). Dicer-Like genes are required for H2O2 and KCl stress responses, pathogenicity and small rna generation in *Valsa mali*. *Front. Microbiol.* 8:1166 10.3389/fmicb.2017.01166 PMC5481355 28690605

[B12] Gao J. Li Y. Ke X. Kang Z. Huang L. (2011). Development of genetic transformation system of *Valsa mali* of apple mediated by PEG. *Act. Microbiol. Sin.* 51 1194–1199. 10.13343/j.cnki.wsxb.2011.09.007 22126074

[B13] Hervas-Aguilar A. Rodriguez J. M. Tilburn J. Arst H. N. Jr. Penalva M. A. (2007). Evidence for the direct involvement of the proteasome in the proteolytic processing of the *Aspergillus nidulans* zinc finger transcription factor PacC. *J. Biol. Chem.* 282 34735–34747. 10.1074/jbc.M706723200 17911112

[B14] Huang W. Shang Y. Chen P. Gao Q. Wang C. (2015). MrpacC regulates sporulation, insect cuticle penetration and immune evasion in *Metarhizium robertsii*. *Environ. Microbiol.* 17 994–1008. 10.1111/1462-2920.12451 24612440

[B15] Ke X. Huang L. Han Q. Gao X. Kang Z. (2013). Histological and cytological investigations of the infection and colonization of apple bark by *Valsa mali* var. *mali*. *Australas. Plant. Pat.* 42 85–93. 10.1007/s13313-012-0158-y

[B16] Ke X. Yin Z. Song N. Dai Q. Voegele R. T. Liu Y. (2014). Transcriptome profiling to identify genes involved in pathogenicity of *Valsa mali* on apple tree. *Fungal Genet. Biol.* 68 31–38. 10.1016/j.fgb.2014.04.004 24747070

[B17] Kramer-Haimovich H. Servi E. Katan T. Rollins J. Okon Y. Prusky D. (2006). Effect of ammonia production by *Colletotrichum gloeosporioides* on pelB activation, pectate lyase secretion, and fruit pathogenicity. *Appl. Environ. Microbiol.* 72 1034–1039. 10.1128/AEM.72.2.1034-1039.2006 16461646 PMC1392887

[B18] Kubicek C. P. Starr T. L. Glass N. L. (2014). Plant cell wall-degrading enzymes and their secretion in plant-pathogenic fungi. *Annu. Rev. Phytopathol.* 52 427–451. 10.1146/annurev-phyto-102313-045831 25001456

[B19] Landraud P. Chuzeville S. Billon-Grande G. Poussereau N. Bruel C. (2013). Adaptation to pH and role of PacC in the rice blast fungus *Magnaporthe oryzae*. *PLoS One* 8:e69236. 10.1371/journal.pone.0069236 23874922 PMC3712939

[B20] Lee D. H. Lee S. W. Choi K. H. Kim D. A. Uhm J. Y. (2006). Survey on the occurrence of apple diseases in Korea from 1992 to 2000. *Plant Pathol. J.* 22 375 10.5423/PPJ.2006.22.4.375

[B21] Livak K. J. Schmittgen T. D. (2001). Analysis of relative gene expression data using real-time quantitative PCR and the 2^-ΔΔC_T_^ Method. *Methods* 25 402–408. 10.1006/meth.2001.1262 11846609

[B22] Ment D. Alkan N. Luria N. Bi F.-C. Reuveni E. Fluhr R. (2015). A role of AREB in the regulation of PACC-dependent acid-expressed-genes and pathogenicity of *Colletotrichum gloeosporioides*. *Mol. Plant Microbe Interact.* 28 154–166. 10.1094/MPMI-09-14-0252-R 25317668

[B23] Merhej J. Richard-Forget F. Barreau C. (2011). The pH regulatory factor Pac1 regulates Tri gene expression and trichothecene production in Fusarium graminearum. *Fungal Genet. Biol.* 48 275–284. 10.1016/j.fgb.2010.11.008 21126599

[B24] Miyara I. Shafran H. Davidzon M. Sherman A. Prusky D. (2010). pH regulation of ammonia secretion by *Colletotrichum gloeosporioides* and its effect on appressorium formation and pathogenicity. *Mol. Plant Microbe Interact* 23 304–316. 10.1094/MPMI-23-3-0304 20121452

[B25] Miyara I. Shafran H. Kramer Haimovich H. Rollins J. Sherman A. Prusky D. (2008). Multi-factor regulation of pectate lyase secretion by Colletotrichum gloeosporioides pathogenic on avocado fruits. *Mol. Plant Pathol.* 9 281–291. 10.1111/j.1364-3703.2007.00462.x 18705870 PMC6640356

[B26] Penalva M. A. Arst H. N. (2002). Regulation of gene expression by ambient pH in Filamentous Fungi and Yeasts. *Microbiol. Mol. Biol.* 66 426–446. 10.1128/mmbr.66.3.426-446.2002 PMC120796 12208998

[B27] Penalva M. A. Espeso E. A. (1996). Three binding sites for the *Aspergillus nidulans* PacC Zinc-finger transcription factor are necessary and sufficient for regulation by ambient pH of the isopenicillin N synthase gene promoter. *J. Biol. Chem.* 271 28825–28830. 10.1074/jbc.271.46.28825 8910527

[B28] Peñalva M. A. Tilburn J. Bignell E. Arst H. N. (2008). Ambient pH gene regulation in fungi: making connections. *Trends Microbiol.* 16 291–300. 10.1016/j.tim.2008.03.006 18457952

[B29] Rollins J. A. (2003). The Sclerotinia sclerotiorum pac1 gene is required for sclerotial development and virulence. *Mol. Plant Microbe Interact.* 16 785–795. 10.1094/MPMI.2003.16.9.785 12971602

[B30] Rollins J. A. Dickman M. B. (2001). pH signaling in Sclerotinia sclerotiorum: identification of a pacC/RIM1 homolog. *Appl. Environ. Microbiol.* 67 75–81. 10.1128/AEM.67.1.75-81.2001 11133430 PMC92519

[B31] Schmidt J. Kricke R. Feige G. B. (2001). Measurements of bark pH with a modified flathead electrode. *Lichenologist* 33 456–460. 10.1006/lich.2001.0341

[B32] Srivastava A. Ohm R. A. Oxiles L. Brooks F. Lawrence C. B. Grigoriev I. V. (2012). A zinc-finger-family transcription factor, AbVf 19, is required for the induction of a gene subset important for virulence in *Alternaria brassicicola*. *Mol. Plant Microbe Interact.* 25 443–452. 10.1094/MPMI-10-11-0275 22185468

[B33] Tamura K. Dudley J. Nei M. Kumar S. (2007). MEGA4: molecular evolutionary genetics analysis (MEGA) software version 4.0. *Mol. Biol. Evol.* 24 1596–1599. 10.1093/molbev/msm092 17488738

[B34] Temme N. Tudzynski P. (2009). Does Botrytis cinerea ignore H2O2-induced oxidative stress during infection? Characterization of Botrytis activator protein 1. *Mol. Plant Microbe Interact.* 22 987–998. 10.1094/MPMI-22-8-0987 19589074

[B35] Tilburn J. Sarkar S. Widdick D. Espeso E. Orejas M. Mungroo J. (1995). The Aspergillus PacC zinc finger transcription factor mediates regulation of both acid-and alkaline-expressed genes by ambient pH. *Embo. J.* 14:779. 10.1002/j.1460-2075.1995.tb07056.x 7882981 PMC398143

[B36] Wang X. Zang R. Yin Z. Kang Z. Huang L. (2014). Delimiting cryptic pathogen species causing apple *Valsa canker* with multilocus data. *Ecol. Evol.* 4 1369–1380. 10.1002/ece3.1030 24834333 PMC4020696

[B37] Wu Y. Xu L. Liu J. Yin Z. Gao X. Feng H. (2017a). A mitogen-activated protein kinase gene (VmPmk1) regulates virulence and cell wall degrading enzyme expression in *Valsa mali*. *Microb. Pathog.* 111 298–306. 10.1016/j.micpath.2017.09.003 28888885

[B38] Wu Y. Xu L. Yin Z. Dai Q. Gao X. Feng H. (2017b). Two members of the velvet family, VmVeA and VmVelB, affect conidiation, virulence and pectinase expression in *Valsa mali*. *Mol. Plant Pathol* 19 1639–1651. 10.1111/mpp.12645 29127722 PMC6638101

[B39] Wubben J. P. ten Have A. van Kan J. A. Visser J. (2000). Regulation of endopolygalacturonase gene expression in *Botrytis cinerea* by galacturonic acid, ambient pH and carbon catabolite repression. *Curr. Genet* 37 152–157. 10.1007/s002940050022 10743572

[B40] Yakoby N. Kobiler I. Dinoor A. Prusky D. (2000). pH Regulation of Pectate Lyase Secretion Modulates the Attack of Colletotrichum gloeosporioides on Avocado Fruits. *Appl. Environ. Microbiol.* 66 1026–1030. 10.1128/aem.66.3.1026-1030.2000 10698767 PMC91938

[B41] Yin Z. Ke X. Huang D. Gao X. Voegele R. T. Kang Z. (2013). Validation of reference genes for gene expression analysis in *Valsa mali* var. *mali using real-time quantitative PCR*. *World J. Microbiol. Biotechnol.* 29 1563–1571. 10.1007/s11274-013-1320-6 23508400

[B42] Yin Z. Liu H. Li Z. Ke X. Dou D. Gao X. (2015). Genome sequence of Valsa canker pathogens uncovers a potential adaptation of colonization of woody bark. *New Phytol.* 208 1202–1216. 10.1111/nph.13544 26137988

[B43] Yu J. H. Hamari Z. Han K. H. Seo J. A. Reyes-Dominguez Y. Scazzocchio C. (2004). Double-joint PCR: a PCR-based molecular tool for gene manipulations in filamentous fungi. *Fungal Genet. Biol.* 41 973–981. 10.1016/j.fgb.2004.08.001 15465386

[B44] Zhang T. Sun X. Xu Q. Candelas L. G. Li H. (2013). The pH signaling transcription factor PacC is required for full virulence in *Penicillium digitatum*. *Appl. Microbiol. Biotechnol.* 97 9087–9098. 10.1007/s00253-013-5129-x 23917633

[B45] Zhou X. Zhang H. Li G. Shaw B. Xu J.-R. (2012). The Cyclase-associated protein Cap1 is important for proper regulation of infection-related morphogenesis in *Magnaporthe oryzae*. *PloS Pathog.* 8:e1002911. 10.1371/journal.ppat.1002911 22969430 PMC3435248

