# Supplementary figures and images for: VmPacC Is Required for Acidification and Virulence in Valsa mali
Source: Front Microbiol. 2018 Aug 23;9:1981. doi: 10.3389/fmicb.2018.01981 (PMC6115506; doi:10.3389/fmicb.2018.01981)

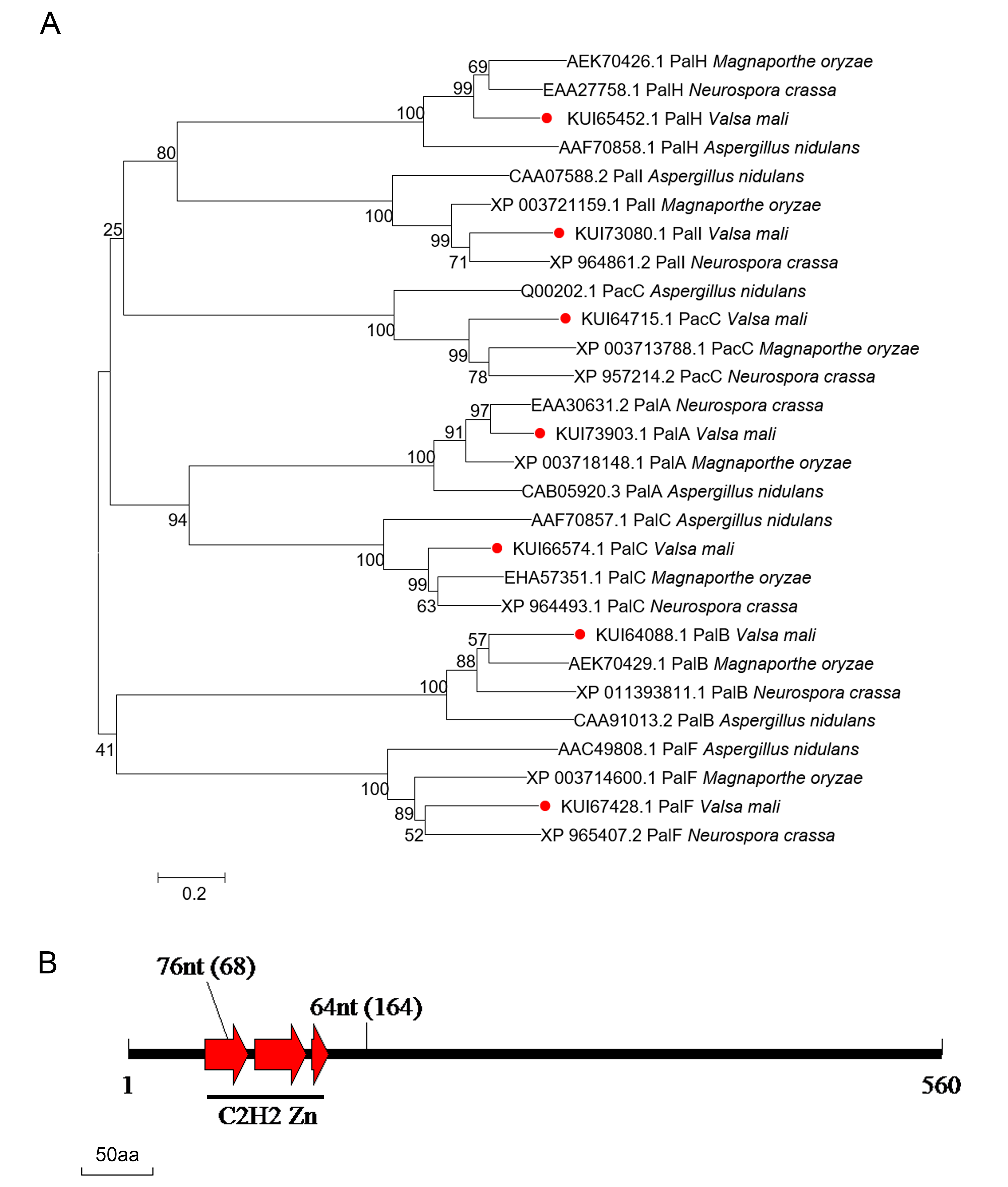

Supplement: FIGURE S1 — Structure and sequence analysis of pH-signalling pathway proteins in Valsa mali. (A) Phylogenetic analysis of pH-signalling pathway proteins of V. mali and their homologs from Magnaporthe oryzae, Aspergillus nidulans, and Neurospora crassa. The amino acid sequences of Pal pathway proteins were analysed using MEGA version 6 and neighbour-joining analysis with 1,000 bootstrap replicates. Numbers on the branches represent the percentage of replicates supporting each branch. Subclades containing pH-signalling pathway proteins of V. mali and orthologs from other species are shaded. The bar represents 20% sequence divergence. (B) The open reading frame (ORF) of VmPacC consists of 1,825 bp, interrupted by two introns, and encodes a predicted protein of 560 amino acids with classical zinc-finger domain and zinc-finger double domain at its N-terminal region. [file Image_1.TIF]

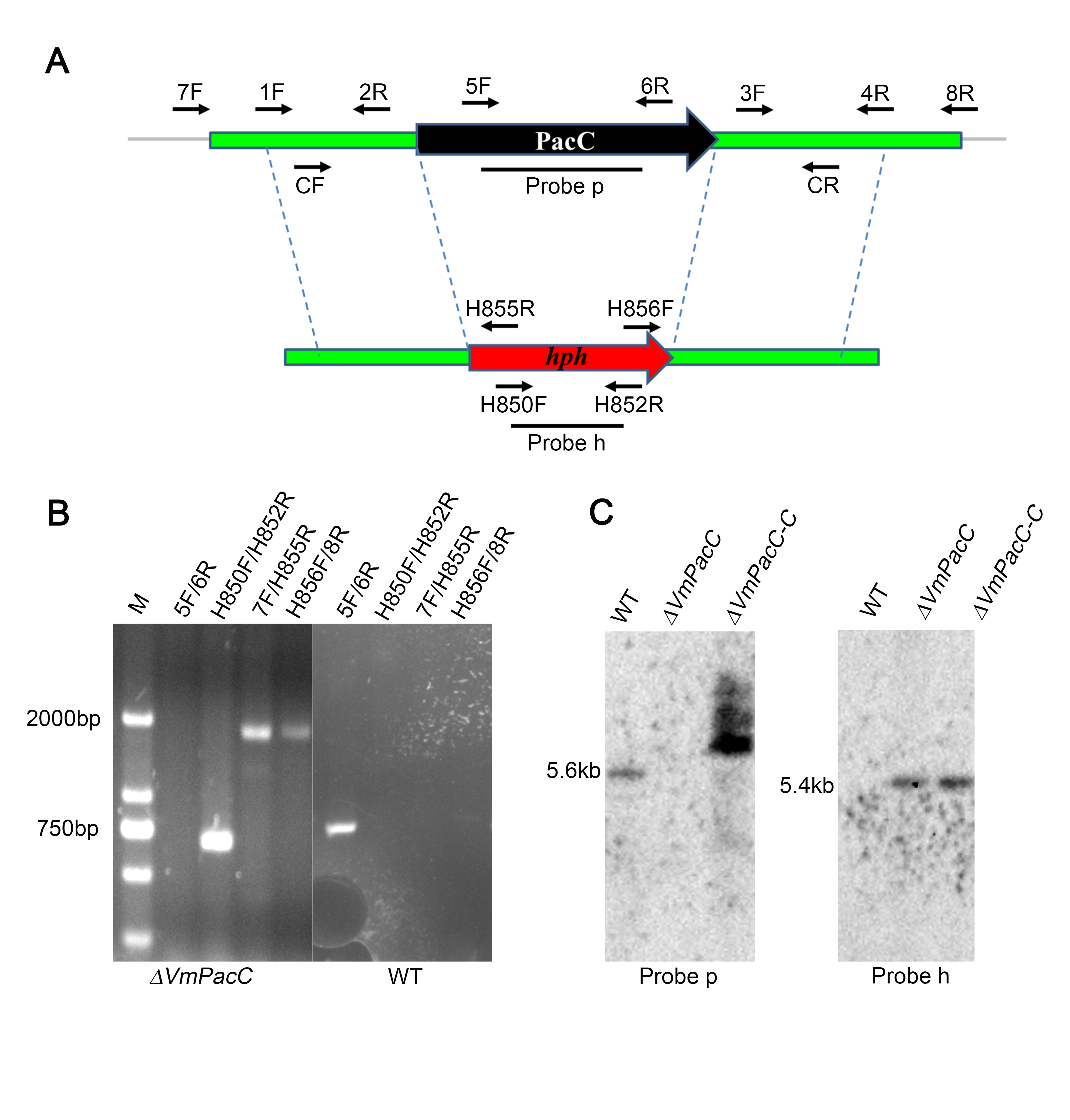

Supplement: FIGURE S2 — Generation of VmPacC gene deletion mutants. (A) Generation of gene replacement constructs. The small arrows mark the position and direction of primers used for PCR. (B) For PCR detection of deletion transformants, four primer pairs (VmPacC-5F/6R, H850F/H852R, VmPacC-7F/H855F, and H856F/VmPacC-8R) were used to detect VmPacC gene deletion mutants. (C) Southern blots of ClaI-digested genomic DNA of wild-type, deletion mutant (ΔVmPacC), and complemented mutant (ΔVmPacC-C) strains hybridised with probe p (VmPacC ORF or probe h (hph ORF)). [file Image_2.TIF]

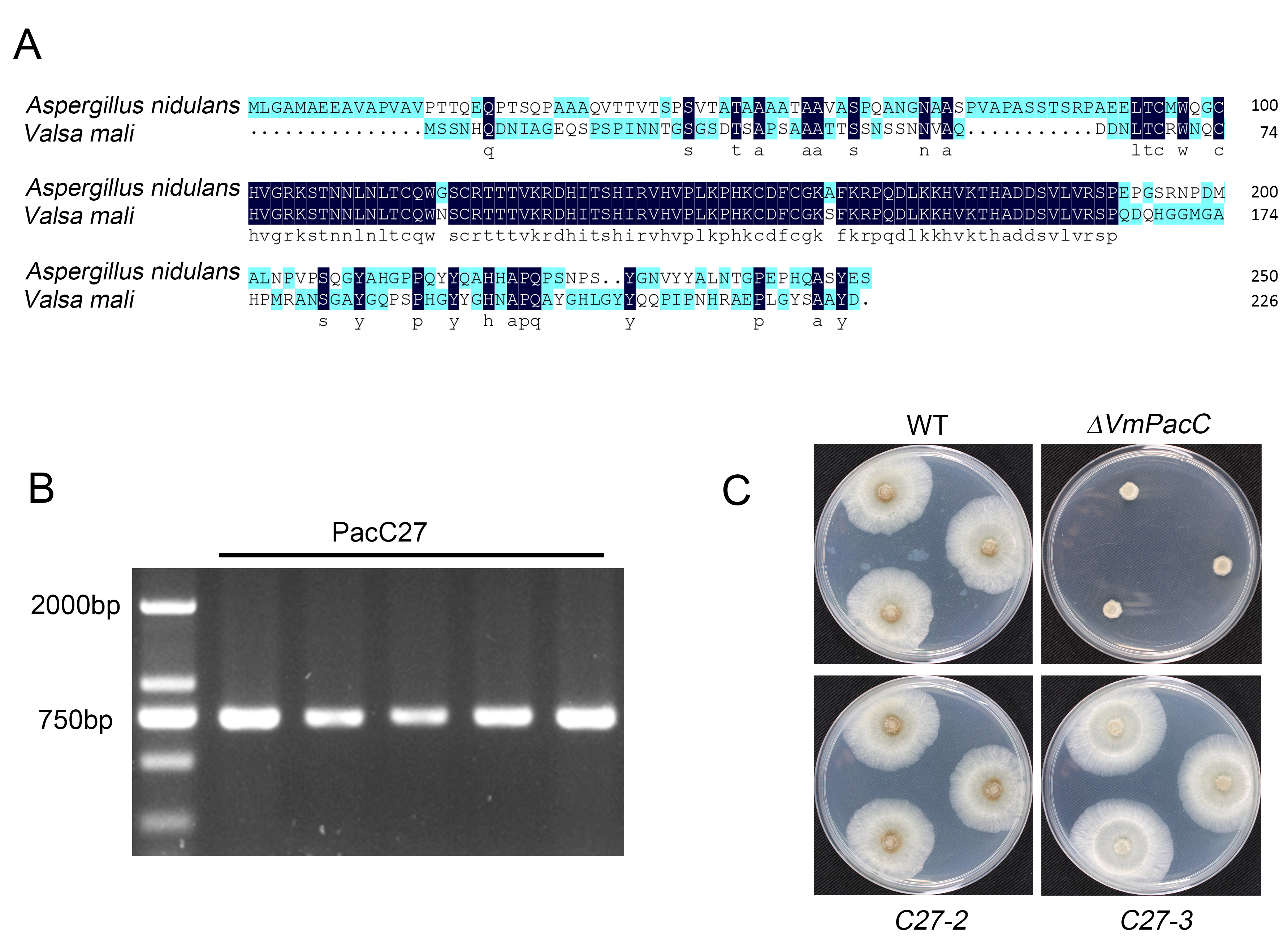

Supplement: FIGURE S3 — Construction of dominant activated mutant of VmPacC27. (A) Prediction of activated sequence with A. nidulans. (B) PCR detection of positive transformants of activated mutants with primer pairs VmPacC27-F/R. (C) Wild-type (WT) deletion and activated mutants grow at pH 9 in unbuffered potato dextrose agar for 3 days. The normal growth was restored in the activated mutants, indicating the VmPacC was expressive. [file Image_3.TIF]
